# Breast cancer incidence and mortality, by age, stage and molecular subtypes, by race/ethnicity in Canada

**DOI:** 10.1093/oncolo/oyae283

**Published:** 2024-11-02

**Authors:** Anna N Wilkinson, Carmina Ng, Larry F Ellison, Jean M Seely

**Affiliations:** Department of Family Medicine, University of Ottawa, Ottawa, Ontario, K1G 5Z3, Canada; Centre for Population Health Data, Statistics Canada, Government of Canada, Ottawa, Ontario, K1A 0T6, Canada; Centre for Population Health Data, Statistics Canada, Government of Canada, Ottawa, Ontario, K1A 0T6, Canada; Department of Radiology, University of Ottawa, Ottawa, Ontario, K1H 8L6, Canada

**Keywords:** breast cancer, race, ethnicity, cancer registry, outcomes, women’s health

## Abstract

**Background:**

Breast cancer (BC) characteristics and outcomes in Canada related to race/ethnicity are not currently documented.

**Methods:**

Age-specific and age-standardized BC incidence and mortality rates, age distribution of cases, proportions of stage, and molecular subtypes were calculated for women aged 20+, by race/ethnicity, using 2006 and 2011 Canadian Census Health and Environment Cohort databases of linked census, cancer, and death data.

**Results:**

In 47 105 BC cases, age-specific incidence rates were higher in Filipina (rate ratio (RR) = 1.27, 95%CI, 1.11-1.46) and multiethnicity (RR = 1.57, 95% CI, 1.18-2.08) compared to White women aged 40-49; and Filipina (RR = 1.16, 95% CI, 1.02-1.31) and Arab (RR = 1.3, 95% CI, 1.02-1.65) women aged 50-59. Median age at diagnosis was 63 among White women and 52-60 among other race/ethnicity groups, with 22.4%-41.1% of cases (*P* < .001) diagnosed before age 50 compared to 16.6% among White women. BC was diagnosed at stage I less frequently among Filipina (38.6%), Black (39.2%), South Asian (40.6%), and First Nations (40.7%) compared to White (46.5%) and Chinese (49.6%) (*P* < .05) women. Black women had higher proportions of BC diagnoses at stages III and IV combined (26.3%) than White women (17.0%, *P* = .001). The proportion of triple-negative BC among Black women (20.5%) was higher than among White (9.5%, *P* < .001). Compared to White, age-specific BC mortality rates were higher among Black women aged 40-49 (RR = 1.4, 1.06-1.85) as well as First Nations (RR = 1.21, 1.01-1.45) and Métis (RR = 1.48, 1.15-1.91) women aged 60-69.

**Interpretation:**

Compared to White women, other Canadian women had an earlier peak age of BC diagnosis and higher proportions of cases diagnosed under age 50. Although many race/ethnicity groups had lower BC incidence and mortality than White, the higher age-specific BC mortality among Black 40-49 and First Nations and Métis women 60-69 merits further investigation.

## Teaser

This article focuses on whether breast cancer characteristics and outcomes differ by race and ethnicity in Canada.

**Implications for practice**

Women from various racial and ethnic groups in Canada are frequently diagnosed with breast cancer at younger ages and more advanced stages than White women. Notably, Black women are more likely to develop aggressive triple-negative breast cancer and experience higher mortality rates at younger ages. Similarly, elevated breast cancer mortality rates have been observed among First Nations and Métis women in their 60s. To address these disparities, earlier screening for these groups could be considered, and targeted research is essential to better understand the underlying causes of breast cancer mortality disparities affecting Black, First Nations, and Métis women.

## Introduction

Breast cancer (BC) is the most common cancer and the second leading cause of death from cancer in Canadian women.^1^ BC outcomes are driven by a complex interplay of biological factors which impact tumor aggressiveness, in combination with other considerations including access to screening and treatment, treatment adherence, socioeconomic status, and comorbidities. These factors can influence BC presentations with earlier age at onset, advanced stage at presentation, more aggressive histologies, and may ultimately lead to increased mortality at earlier ages.

BC outcomes have been reported to vary by race/ethnicity. In the United States, Black, Hispanic, Asian American/Pacific Islander, and Native American women are respectively 45%, 93%, 83%, and 46% more likely than White women to be diagnosed with BC under the age of 50.^2,3^ Together, these women are 24% more likely to be diagnosed with advanced stage BC than White women. BC mortality is 40% higher among Black women compared to White women, despite a lower incidence.^4^ BC mortality among White women has decreased by 40% from 1990 to 2017, but only by 26% among Black women,^5^ and Black women under 50 have 2.11 times the BC death rate of White women.^2^ Poorer BC outcomes have been observed among Black women independent of socioeconomic status.^6^ While race/ethnicity data are collected for cancer registry cases in the United States, this information is not available in Canadian registries.^7-9^

In 2021, 1 in 4 Canadians (26.5%) identified as groups other than White, 2 times higher than was noted in 2001.^10^ It is unknown if BC characteristics and outcomes differ by race/ethnicity in Canada as they do in the United States. By linking the Canadian Cancer Registry (CCR) and census data, the aim of our study was to describe BC characteristics by race/ethnicity, including incidence and mortality rates by age, stage, and molecular subtypes.

## Methods

This study was a secondary analysis of nationally de-identified data collected by Statistics Canada, and as such, ethics approval was not required. The 2006 Canadian Census Health and Environment Cohort (CanCHEC) (*n* = 5.9 million) and 2011 CanCHEC (*n* = 6.5 million) were used.^11^ Race/ethnicity information from the 2006 long-form Census,^12^ conducted May 16, 2006, and the 2011 National Household Survey (NHS),^13^ conducted May 10, 2011, were linked to BC cases in the CCR,^9^ diagnosed from census/NHS day up to December 31, 2015, and deaths in the Canadian Vital Statistics Database (CVSD),^14^ up to December 31, 2019. The CCR is a population-based database comprised of data annually collected and reported to Statistics Canada by each provincial and territorial cancer registry. As no cancer data for Québec was available after 2010, follow-up for Québec residents ended on or before December 31, 2010 for incidence. Yukon mortality data was not available after 2016, hence BC mortality follow-up for these residents ended on or before December 31, 2016. BC cases were identified according to the International Classification of Diseases for Oncology, 3rd edition.^15^ Male BC cases were excluded. BC deaths were identified according to the International Classification of Diseases, Tenth Revision, codes C50.0-C50.9.^16^

Race/ethnicity was derived using the visible minority and Indigenous identities concepts common to both 2006 Census^17^ and 2011 NHS,^18^ where respondents were asked to mark applicable categories. The 2006 and 2011 cohorts were pooled for BC incidence and mortality analyses, with duplicate cohort members removed (*n* = 1.2 million). The 2006 long-form Census was distributed to 20% of households (response rate 93.8%) and the 2011 NHS was distributed to 33% of households (response rate 68.6%). While 100% of households in First Nations, remote areas, and northern communities were asked to complete the survey, certain communities did not participate.^19,20^ In general, Indigenous persons were sampled at a higher rate compared to other groups.

Females aged ≥20 years on census/NHS day were included. Person-years were accumulated from census/NHS day to date of BC diagnosis (for incidence analyses), date of death, or end of follow-up (maximum December 31, 2015 for incidence and December 31, 2019 for mortality), whichever was earliest. BC age-standardized incidence rates (ASIR) and age-standardized mortality rates (ASMR) were calculated for race/ethnicity groups, using the World Health Organization’s world standard population.^21^ Age-specific BC incidence and mortality rates were calculated for ages 30-39, 40-49, 50-59, 60-69, 70-79, and 80+. Rates, rate ratios (RR), and 95% CI were calculated for each race/ethnicity category with at least 10 BC cases/deaths. The White group was used as the reference category for all analyses.

The proportion of BC cases at each age at diagnosis and of BC deaths at each age at death were calculated by race/ethnicity groups. These proportions were then smoothed using local regression (loess) and age distributions were plotted. Peak age at BC diagnosis and death were determined for categories with at least 50 cases/deaths. Proportions of BC diagnosed at ages 20-49 were also compared.

The 2011 cohort was used for BC stage and molecular subtype analyses as information on stage was not reported to the CCR prior to 2010. The American Joint Committee on Cancer 7th edition staging manual was used to classify stage at diagnosis for BC cases diagnosed May 10, 2011 to December 31, 2015.^22^ Proportions of stage were compared between race/ethnicity groups with at least 10 BC cases per stage, using White as the reference category.

The molecular subtype analysis included BC cases diagnosed from January 1, 2012—the start of the period for which the variables necessary to determine molecular subtype were consistently reported to the CCR—to December 31, 2015. As gene expression profiling information was not available in the CCR, the status of immunohistochemical elements, including human epidermal growth factor receptor 2 (HER2), estrogen receptor (ER), progesterone receptor (PR), and grade were used to approximate molecular subtype. BC cases were divided into five molecular subtypes—luminal A (LA), luminal B (LB), luminal B-like (LB-like), HER2+ and triple-negative (TN). Subtypes were defined as follows: LA (ER positive and PR positive, HER2 negative and low/intermediate grade on the Nottingham or Bloom-Richardson grading scale); LB (ER and PR positive, HER2 negative and high grade, or, ER positive and PR negative, HER2 negative and any grade); LB-like (ER positive and PR positive or negative, HER2 positive, any grade); HER2+ (ER negative, PR negative, HER2 positive); and TN (ER negative, PR negative, HER2 negative).^23-25^ BC cases with missing information were classified as unknown. Proportions of subtypes were compared between race/ethnicity groups with at least 10 cases for each subtype, using White as the reference category.

Two-sided chi-squared tests, with a significance level of 0.05, were used to test the null hypothesis of no difference in proportions.

## Results

### Incidence

The incidence analyses included 3 801 340 women, with 47 105 BC cases identified during follow-up. The median age of White women was 49 years, while the median age of all other racial and ethnic groups ranged from 37 to 45 years (Table 1). BC ASIR and age-specific incidence rates are presented in Tables 1 and 2.

**Table 1.** Incidence analysis sample description, median and mean (SD) age of sample, age at breast cancer diagnosis, peak age at breast cancer diagnosis, age-standardized breast cancer incidence rates, and rate ratios (reference = White).

Race/ethnicity	*N*	Median age of sample	Mean age of sample (SD)	Breast cancer cases	Median age at diagnosis	Mean age at diagnosis (SD)	Peak age at diagnosis	Age-standardized incidence rate, per 100 000 person-years (95% CI)	Age-standardized incidence rate ratio (95% CI)
Arab	23 450	38	40.3 (14.3)	205	53	54.9 (12.5)	48	135.7 (117.2-156.8)	1.00 (0.87-1.15)
Black	77 185	40	42.7 (15.5)	605	56	57.1 (13.6)	50	102.5 (94.5-111.3)	0.76 (0.70-0.82)
Chinese	170 255	45	45.9 (16.2)	1620	56	57.6 (13.2)	50	110.6 (105.1-116.4)	0.82 (0.77-0.86)
Filipina	69 780	42	43.8 (14.3)	785	54	55.8 (11.8)	50	142.7 (132.6-153.9)	1.05 (0.98-1.13)
First Nations	134 860	40	41.6 (15.0)	1335	57	57.9 (12.2)	55	124.3 (117.6-131.3)	0.92 (0.87-0.97)
Inuit	11 350	37	39.1 (14.3)	60	53.5	55.3 (12.0)	42	70.6 (53.4-92.1)	0.52 (0.40-0.67)
Japanese	11 745	43	47.1 (17.3)	155	60	59.7 (14.1)	60	148.2 (124.9-178.5)	1.09 (0.93-1.29)
Korean	17 830	42	42.7 (14.4)	125	52	52.9 (11.9)	48	91.2 (75.3-110.6)	0.67 (0.56-0.81)
Latin American	35 395	40	41.4 (14.1)	220	56	56.4 (11.9)	56	89.7 (78.0-103.3)	0.66 (0.58-0.76)
Multiethnicity	14 775	40	42.0 (15.3)	165	55	55.6 (12.8)	51	149.4 (127.1-175.4)	1.10 (0.94-1.29)
Métis	47 845	42	42.6 (14.9)	460	58	59.0 (12.5)	57	120.1 (109.3-132.1)	0.89 (0.81-0.97)
Other ethnicity	10 960	42	43.6 (15.5)	95	59	58.5 (13.1)	57	111.0 (89.8-137.7)	0.82 (0.67-1.00)
South Asian	157 705	40	42.8 (15.5)	1270	57	57.8 (12.9)	55	104.9 (99.2-111.0)	0.77 (0.73-0.82)
Southeast Asian	27 365	41	42.3 (14.9)	145	56	57.0 (14.2)	54	76.3 (64.0-90.9)	0.56 (0.48-0.67)
West Asian	18 960	40	41.2 (14.5)	165	52	53.2 (11.2)	50	120.1 (102.0-141.3)	0.89 (0.76-1.04)
White	2 971 880	49	49.6 (17.3)	39 695	63	63.2 (13.4)	65	135.7 (134.3-137.2)	reference
Total	3 801 340	47	48.2 (17.1)	47 105	62	62.3 (13.5)			

2006 and 2011 CanCHEC female respondents aged 20+, race/ethnicity from 2006 long-form Census of Canada and 2011 National Household Survey, follow-up of cancer in the Canadian Cancer Registry up to 2015 (up to 2010 for Québec). Rates are age-standardized to the World Health Organization's World Standard Population.

Abbreviations: CanCHEC, Canadian Census Health and Environment Cohort; SD, Standard Deviation.

**Table 2.** Age-specific breast cancer incidence and mortality rates and rate ratios (reference = White).

Race/ethnicity	Age group	Crude incidence rate, per 100 000 person-years (95% CI)	Crude incidence rate ratio (95% CI)	Crude mortality rate, per 100 000 person-years (95% CI)	Crude mortality rate ratio (95% CI)
Arab	30-39	38.5 (19.7-57.4)	1.04 (0.63-1.70)		
	40-49	168.7 (126.0-211.3)	1.23 (0.96-1.59)	16.1 (8.0-24.2)	1.05 (0.63-1.75)
	50-59	286.5 (217.9-355.1)	1.30 (1.02-1.65)	35.1 (20.1-50.1)	1.08 (0.70-1.66)
	60-69	272.2 (177.9-366.5)	0.79 (0.56-1.12)	49.7 (24.5-74.8)	0.93 (0.56-1.54)
	70-79	251.8 (128.4-375.2)	0.64 (0.39-1.04)		
	80+			153.5 (66.6-240.3)	0.88 (0.50-1.55)
Black	30-39	45.7 (33.8-57.5)	1.23 (0.94-1.60)		
	40-49	108.0 (90.0-126.0)	0.79 (0.67-0.93)	21.4 (15.6-27.2)	1.40 (1.06-1.85)
	50-59	174.8 (147.0-202.6)	0.79 (0.67-0.93)	37.1 (28.2-46.0)	1.14 (0.90-1.46)
	60-69	230.5 (191.9-269.1)	0.67 (0.56-0.79)	56.2 (42.8-69.7)	1.05 (0.83-1.34)
	70-79	284.4 (225.0-343.9)	0.72 (0.59-0.89)	68.4 (48.8-87.9)	0.77 (0.58-1.03)
	80+	246.1 (163.4-328.8)	0.68 (0.49-0.96)	139.9 (99.5-180.4)	0.80 (0.60-1.07)
Chinese	30-39	36.3 (28.2-44.4)	0.98 (0.78-1.23)		
	40-49	151.1 (136.5-165.7)	1.10 (1.00-1.22)	14.1 (10.6-17.7)	0.92 (0.71-1.20)
	50-59	197.3 (179.7-214.8)	0.89 (0.82-0.98)	18.0 (14.0-22.1)	0.56 (0.44-0.70)
	60-69	239.3 (213.7-264.9)	0.69 (0.62-0.77)	30.7 (24.0-37.3)	0.57 (0.46-0.71)
	70-79	225.8 (194.0-257.7)	0.57 (0.50-0.66)	32.7 (23.4-41.9)	0.37 (0.28-0.49)
	80+	199.5 (162.7-236.2)	0.55 (0.46-0.67)	69.1 (53.3-84.8)	0.40 (0.31-0.50)
Filipina	30-39	36.0 (24.6-47.5)	0.97 (0.70-1.34)		
	40-49	174.1 (150.9-197.2)	1.27 (1.11-1.46)	15.1 (9.8-20.4)	0.99 (0.69-1.41)
	50-59	255.5 (223.5-287.5)	1.16 (1.02-1.31)	36.7 (27.7-45.7)	1.13 (0.88-1.45)
	60-69	326.5 (277.9-375.2)	0.95 (0.81-1.10)	39.9 (27.5-52.3)	0.75 (0.55-1.02)
	70-79	344.2 (270.6-417.8)	0.87 (0.71-1.08)	62.5 (40.1-84.9)	0.70 (0.49-1.01)
	80+	205.9 (125.2-286.6)	0.57 (0.39-0.85)	71.1 (37.3-104.9)	0.41 (0.25-0.66)
First Nations	30-39	28.6 (21.9-35.3)	0.77 (0.61-0.98)		
	40-49	107.4 (94.4-120.4)	0.78 (0.69-0.89)	15.9 (12.0-19.9)	1.04 (0.81-1.34)
	50-59	224.6 (203.1-246.2)	1.02 (0.92-1.12)	33.6 (27.3-40.0)	1.04 (0.85-1.26)
	60-69	344.5 (308.5-380.4)	1.00 (0.90-1.11)	64.7 (53.3-76.2)	1.21 (1.01-1.45)
	70-79	341.2 (289.6-392.8)	0.87 (0.74-1.01)	96.9 (76.6-117.3)	1.09 (0.88-1.35)
	80+	306.9 (229.3-384.6)	0.85 (0.66-1.10)	154.1 (113.7-194.5)	0.88 (0.68-1.15)
Inuit	40-49	92.3 (51.9-132.8)	0.67 (0.43-1.05)		
	50-59	128.9 (69.4-188.5)	0.58 (0.37-0.93)		
Japanese	40-49	172.8 (113.8-231.8)	1.26 (0.90-1.78)		
	50-59	272.2 (180.7-363.6)	1.23 (0.88-1.73)		
	60-69	371.6 (256.5-486.8)	1.08 (0.79-1.47)		
	70-79	326.8 (196.0-457.5)	0.83 (0.56-1.24)	77.9 (29.6-126.2)	0.88 (0.47-1.63)
	80+	267.8 (132.3-403.3)	0.74 (0.45-1.23)		
Korean	40-49	125.0 (86.3-163.8)	0.91 (0.67-1.25)		
	50-59	152.9 (104.9-200.8)	0.69 (0.51-0.95)		
	60-69	207.7 (126.3-289.2)	0.60 (0.41-0.89)		
Latin American	40-49	86.9 (63.0-110.7)	0.63 (0.48-0.84)		
	50-59	165.1 (127.0-203.3)	0.75 (0.59-0.94)	27.8 (17.1-38.4)	0.86 (0.58-1.26)
	60-69	260.1 (193.2-327.0)	0.75 (0.58-0.98)	49.7 (30.6-68.8)	0.93 (0.63-1.37)
Multiethnicity	40-49	214.3 (153.7-275.0)	1.57 (1.18-2.08)		
	50-59	215.8 (149.8-281.9)	0.98 (0.72-1.33)	32.5 (13.3-51.8)	1.00 (0.55-1.81)
	60-69	360.9 (244.6-477.2)	1.05 (0.76-1.44)	54.6 (22.3-86.9)	1.02 (0.57-1.85)
	70-79	341.6 (179.2-504.0)	0.87 (0.54-1.40)		
Métis	30-39	31.7 (19.0-44.3)	0.85 (0.57-1.28)		
	40-49	96.1 (74.6-117.5)	0.70 (0.56-0.88)	10.3 (4.7-15.9)	0.67 (0.39-1.16)
	50-59	205.3 (172.1-238.5)	0.93 (0.79-1.09)	26.8 (17.6-35.9)	0.83 (0.59-1.16)
	60-69	296.8 (242.8-350.8)	0.86 (0.72-1.03)	79.2 (59.2-99.2)	1.48 (1.15-1.91)
	70-79	398.6 (300.2-497.1)	1.01 (0.79-1.30)	100.5 (65.7-135.3)	1.13 (0.80-1.60)
	80+	489.3 (314.2-664.4)	1.36 (0.95-1.94)	234.6 (147.7-321.5)	1.35 (0.93-1.95)
Other ethnicity	40-49	122.1 (69.9-174.4)	0.89 (0.58-1.37)		
	50-59	157.6 (91.7-223.4)	0.71 (0.47-1.08)		
	60-69	262.6 (157.6-367.7)	0.76 (0.51-1.14)		
	70-79	433.8 (238.7-628.9)	1.10 (0.70-1.73)		
South Asian	30-39	32.3 (25.6-38.9)	0.87 (0.70-1.08)		
	40-49	104.7 (92.0-117.5)	0.77 (0.68-0.87)	8.3 (5.5-11.0)	0.54 (0.39-0.76)
	50-59	201.4 (180.5-222.3)	0.91 (0.82-1.01)	22.5 (17.3-27.8)	0.69 (0.55-0.88)
	60-69	259.1 (230.8-287.4)	0.75 (0.67-0.84)	35.0 (27.2-42.8)	0.66 (0.52-0.82)
	70-79	249.9 (212.3-287.4)	0.63 (0.55-0.74)	41.1 (30.0-52.3)	0.46 (0.35-0.61)
	80+	240.6 (183.4-297.7)	0.67 (0.53-0.85)	81.1 (57.9-104.3)	0.47 (0.35-0.62)
Southeast Asian	40-49	73.0 (48.1-97.9)	0.53 (0.38-0.75)		
	50-59	102.8 (70.1-135.5)	0.47 (0.34-0.64)	13.7 (5.2-22.1)	0.42 (0.23-0.78)
	60-69	219.9 (147.0-292.7)	0.64 (0.46-0.89)		
	70-79	177.1 (87.5-266.8)	0.45 (0.27-0.75)		
	80+			89.0 (33.8-144.2)	0.51 (0.27-0.95)
West Asian	40-49	163.1 (118.8-207.4)	1.19 (0.91-1.57)		
	50-59	231.0 (170.5-291.5)	1.05 (0.80-1.36)		
	60-69	263.5 (169.2-357.7)	0.76 (0.53-1.09)		
	70-79			104.6 (42.8-166.4)	1.18 (0.65-2.13)
White	30-39	37.1 (35.0-39.3)			
	40-49	136.9 (133.2-140.6)		15.3 (14.4-16.3)	
	50-59	220.8 (216.3-225.3)		32.5 (31.2-33.7)	
	60-69	344.9 (338.5-351.4)		53.4 (51.7-55.2)	
	70-79	393.6 (384.9-402.4)		88.7 (85.8-91.6)	
	80+	360.7 (350.7-370.6)		174.4 (169.6-179.3)	

2006 and 2011 CanCHEC female respondents aged 20+, race/ethnicity from 2006 long-form Census of Canada and 2011 National Household Survey, follow-up of cancer in the Canadian Cancer Registry up to 2015 (up to 2010 for Québec); follow-up of deaths in the Canadian Vital Statistics Database up to 2019 (up to 2016 for Yukon). Rates reported when ≥10 cases/deaths.

Abbreviations: CanCHEC, Canadian Census Health and Environment Cohort.

Examining rates for women younger than 50, significantly lower age-specific BC incidence rates were observed among First Nations (RR = 0.77, 95% CI, 0.61-0.98) women for the age group 30-39, compared with White women (Table 2). Higher age-specific incidence was observed among Filipina (RR = 1.27, 95% CI, 1.11-1.46) and multiethnicity (RR = 1.57, 95% CI, 1.18-2.08) women aged 40-49. In this same age group, Southeast Asian (RR = 0.53, 95% CI, 0.38-0.75), Latin American (RR = 0.63, 95% CI, 0.48-0.84), Métis (RR = 0.70, 95% CI, 0.56-0.88), South Asian (RR = 0.77, 95% CI, 0.68-0.87), First Nations (RR = 0.78, 95% CI, 0.69-0.89), and Black (RR = 0.79, 95% CI, 0.67-0.93) women had significantly lower age-specific incidence rates compared to White women.

Among age groups 50 and older, significantly higher incidence rates were observed for Filipina (RR = 1.16, 95% CI, 1.02-1.31) and Arab (RR = 1.30, 95% CI, 1.02-1.65) women aged 50-59 compared to White women. In women 50 and older, age-specific incidence rates for White women were generally higher than other race/ethnicity groups, or no significant differences in rates were observed.

### Age at diagnosis

**Figure 1.**
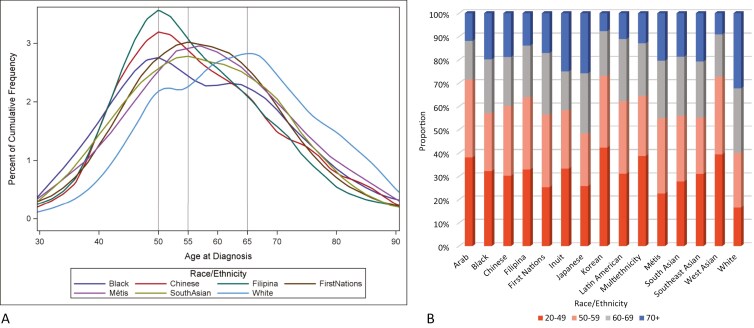
Age at breast cancer diagnosis, by race/ethnicity, Canada. (A) Distributions of age at breast cancer diagnosis, by race/ethnicity, for groups ≥400 cases. (B) Distributions of age at breast cancer diagnosis, proportions, by race/ethnicity. Note: 2006 and 2011 CanCHEC female respondents aged 20+, race/ethnicity from 2006 long-form Census of Canada and 2011 National Household Survey, follow-up of cancer in the Canadian Cancer Registry up to 2015 (up to 2010 for Québec). CanCHEC, Canadian Census Health and Environment Cohort.

The median age at BC diagnosis was 63 years for White women and ranged from 52 to 60 for other race/ethnicity groups (Table 1). The peak age at BC diagnosis was 65 for White women and varied from 42 to 60 for other groups (Table 1) (Figure 1A). Inuit women had the earliest peak age of diagnosis at age 42, while Arab and Korean peaked at age 48 and Black, West Asian, Chinese and Filipina women at age 50. The proportion of cases diagnosed before the age of 50 ranged from 22.4% of BC cases among Métis to 41.1% of cases among Korean women (Figure 1B). Significantly higher proportions (22.4%-41.1%, *P* < .001) of cases were diagnosed before age 50 for all groups compared to White women (16.6%).

### Stage at diagnosis

**Figure 2.**
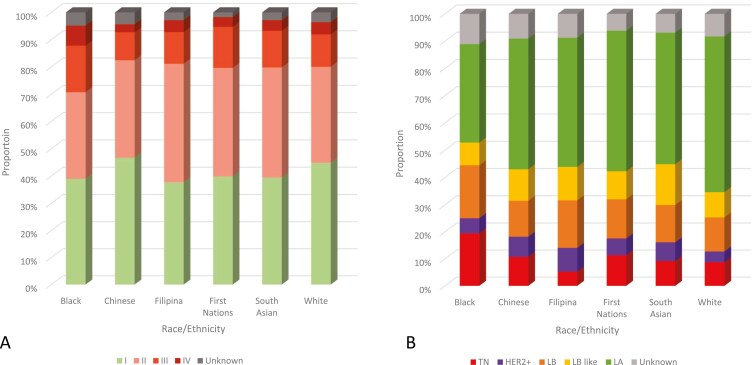
(A) Proportions of stage at breast cancer diagnosis by race/ethnicity. (B) Proportions of molecular subtypes of breast cancer, by race/ethnicity. Notes: 2011 CanCHEC female respondents aged 20+, race/ethnicity from 2011 National Household Survey, follow-up of cancer in the Canadian Cancer Registry up to 2015 for stage and 2012-2015 for subtype. Québec not included. Proportions for groups with at least 10 BC cases per stage/subtype. CanCHEC, Canadian Census Health and Environment Cohort HER2+, human epidermal growth factor receptor 2 positive; LA, luminal A; LB, luminal B; LB-like, luminal B-like; TN, triple-negative

The analysis by stage at diagnosis included 15 260 BC cases from 6 race/ethnicity groups (Figure 2A). Excluding cases with unknown stage (3.6%), almost half of BC cases were diagnosed at stage I among both White (46.5%) and Chinese (49.6%) women, while lower proportions of cases were diagnosed at stage I among Filipina (38.6%, *P* = .005), Black (39.2%, *P* = .042), South Asian (40.6%, *P* = .009), and First Nations (40.7%, *P* = .008) women. Combining stages III and IV BC, a higher proportion of BC cases among Black women were diagnosed at these advanced stages (26.3%, *P* = .001) compared to White women (17.0%), while a lower proportion was observed among Chinese women (13.2%, *P* = .012). In women under age 50, 21.0% of BC cases were diagnosed at stage III or IV among White women, compared with 49.0% (*P* < .001) among Black, 38.4% (*P* < .001) among Chinese, 33.9% (*P* = .030) among Filipina, 33.7% (*P* = .008) among South Asian, and 26.0% (*P* = 0.269) among First Nations women.

### Molecular subtypes

The analysis by molecular subtype included 13 130 BC cases from 6 race/ethnicity groups (Figure 2B). Excluding cases of unknown subtype (8.3%), LA comprised 62.3% of BC cases among White women; however, compared to White, proportions of LA were lower among Black (37.9%, *P* < .001), Filipina (51.7%, *P* = .001), South Asian (52.0%, *P* < .001), Chinese (53.2%, *P* < .001), and First Nations (55.2%, *P* = .003) women. The proportion of TN BC among Black women (20.5%) was higher than that among White women (9.5%, *P* < .001), while a lower proportion of TN BC was observed among Filipina (5.4%, *P* = .024) compared with White women.

### Mortality

**Figure 3.**
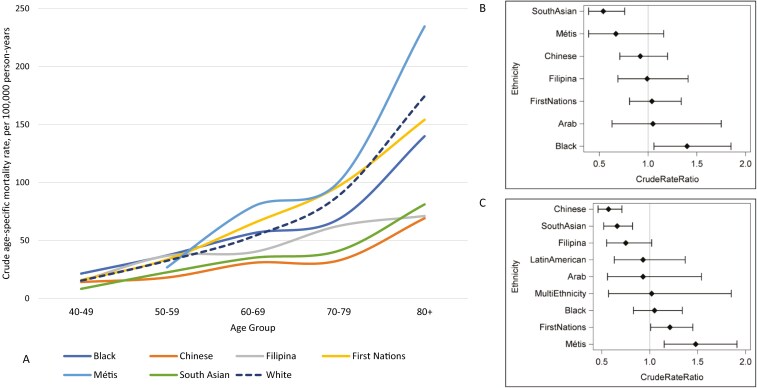
(A) Age-specific breast cancer mortality rates, by race/ethnicity. (B) Crude age-specific mortality rate ratios, age group 40-49 by race/ethnicity. (C) Crude age-specific mortality rate ratios, age group 60-69 by race/ethnicity. Notes: 2006 and 2011 CanCHEC female respondents aged 20+, race/ethnicity from 2006 long-form Census of Canada and 2011 National Household Survey, follow-up of deaths in the Canadian Vital Statistics Database up to 2019 (up to 2016 for Yukon). Groups with ≥10 deaths included. CanCHEC = Canadian Census Health and Environment Cohort.

The BC mortality analysis included 4 313 210 women, with 18 220 BC deaths identified during followup. ASMRs are presented in Table 3. Examining age-specific BC mortality, higher mortality was observed among Black women ages 40-49 (RR = 1.40, 95% CI, 1.06-1.85) as well as among First Nations (RR = 1.21, 95% CI, 1.01-1.45) and Métis women ages 60-69 (RR = 1.48, 95% CI, 1.15-1.91) compared to White women (Table 2) (Figure 3A, 3B, and 3C). Among the groups with sufficient data for comparisons, notably lower BC mortality was observed among Chinese (50-59 to 80+ age groups) and South Asian women (40-49 to 80+ age groups). The median age at BC death among White and Japanese women was 71, whereas it was 64 or younger for women of other race/ethnicity groups examined. The peak age at BC death for White women was 79, while for Métis, First Nations, Latin American, South Asian, Chinese, Black, Filipina, and Arab women the peak ages were 65, 62, 62, 60, 58, 56, 55, and 54, respectively (Table 3).

**Table 3.** Mortality analysis sample description, median and mean (SD) age of sample, age at breast cancer death, peak age at breast cancer death, age-standardized breast cancer mortality rates and rate ratios (reference = White).

Race/ethnicity	*N*	Median age of sample	Mean age of sample (SD)	Breast cancer deaths	Median age at death	Mean age at death (SD)	Peak age at death	Age-standardized mortality rate, per 100 000 person-years (95% CI)	Age-standardized mortality rate ratio (95% CI)
Arab	32 410	38	40.0 (14.0)	75	57.5	60.4 (15.8)	54	21.0 (16.4-27.1)	0.93 (0.74-1.18)
Black	90 515	40	42.5 (15.5)	295	61	62.2 (15.4)	56	23.7 (21.0-26.8)	1.05 (0.93-1.18)
Chinese	1,75 800	44	45.8 (16.2)	350	62	64.3 (15.2)	58	12.3 (11.0-13.9)	0.55 (0.49-0.61)
Filipina	72 250	42	43.8 (14.3)	185	58	60.9 (12.6)	55	18.0 (15.5-21.4)	0.80 (0.69-0.92)
First Nations	1,39 610	40	41.8 (15.1)	455	63	63.0 (13.8)	62	24.2 (22.0-26.6)	1.07 (0.98-1.18)
Inuit	11 810	37	39.2 (14.3)	10	55	56.4 (10.4)		8.4 (4.4-15.9)	0.37 (0.21-0.65)
Japanese	12 075	43	47.0 (17.3)	30	71	69.8 (15.2)		15.9 (10.6-28.7)	0.70 (0.49-1.01)
Korean	18 220	42	42.6 (14.4)	25	61	60.7 (13.5)		11.0 (7.2-17.9)	0.49 (0.33-0.72)
Latin American	43 085	39	41.3 (14.0)	75	62	62.8 (12.3)	62	14.1 (11.0-18.3)	0.62 (0.49-0.79)
Multiethnicity	15 690	40	42.0 (15.3)	40	59	58.3 (12.7)		18.6 (13.0-27.0)	0.82 (0.59-1.14)
Métis	50 285	42	42.8 (14.9)	170	64	65.6 (12.8)	65	24.7 (21.1-29.2)	1.10 (0.94-1.28)
Other ethnicity	11 560	42	43.5 (15.5)	25	64	62.4 (16.5)		15.8 (10.2-26.3)	0.70 (0.47-1.04)
South Asian	1,62 120	40	42.8 (15.5)	295	62	63.6 (14.4)	60	13.0 (11.6-14.8)	0.58 (0.51-0.65)
Southeast Asian	31 275	41	42.6 (15.0)	40	63	65.3 (15.5)		8.5 (5.9-12.8)	0.38 (0.27-0.52)
West Asian	20 270	39	41.1 (14.5)	40	63	63.1 (15.1)		17.0 (12.2-24.1)	0.75 (0.55-1.03)
White	34,26 235	49	49.6 (17.3)	16 110	71	70.5 (14.4)	79	22.5 (22.2-23.0)	reference
Total	43,13 210	47	48.2 (17.1)	18 220	70	69.6 (14.6)			

2006 and 2011 CanCHEC female respondents aged 20+, race/ethnicity from 2006 long-form Census of Canada and 2011 National Household Survey, follow-up of deaths in the Canadian Vital Statistics Database up to 2019 (up to 2016 for Yukon). Peak age at death reported when ≥50 deaths. Rates are age-standardized to the World Health Organization's World Standard Population.

Abbreviations: CanCHEC, Canadian Census Health and Environment Cohort.

## Interpretation

The results of this study show that BC outcomes in Canada vary by race/ethnicity. Compared to White women, who have an older age distribution in Canada, other women had an earlier peak age at BC diagnosis and higher proportions of BC cases diagnosed before the age of 50. Among the 6 groups with sufficient cases staged, Black, Filipina, First Nations, and South Asian women had a greater proportion of advanced stage disease (stages II, III, and IV) at diagnosis compared to White and Chinese, and over a quarter of BC cases were diagnosed at stages III or IV among Black women. White women had a higher proportion of the better prognosis LA subtype than the 5 other race/ethnicity groups compared for BC subtypes, and the proportion of TN BC was highest in Black women. Inuit, Southeast Asian, Korean, Chinese, South Asian, Latin American, and Filipina women had a significantly lower ASMR than White women. Although Black women aged 40-49 experienced a lower incidence of BC than White women, their corresponding mortality rate was 40% higher. Among Métis and First Nations women aged 60-69, age-specific BC mortality rates were 50% and 20% higher than that of White women, respectively. White and Japanese women had a median age at death from BC of 71, while the median age at death from BC of other women was at least 7 years younger.

This study confirms that Canadian women of all race/ethnicity groups other than White generally have an earlier peak age at BC diagnosis as has been seen in other countries.^2,3^ The median age of BC diagnosis among Black women (56) in Canada was comparable, and in fact lower than that seen in the United States (60), and the median age at diagnosis among Canadian White women was also comparable to that noted in the United States (63 and 64, respectively).^26,27^ The percentage of BC cases diagnosed below the age of 50 in Black women was greater in Canada (32.2%) compared to the US (22%), and was also slightly higher in White women (16.6% and 14%, respectively). The earlier age of diagnosis among women other than White may be partially explained by the younger population structure of most race/ethnicity groups in Canada compared to White.^28^ Initiation of BC screening at age 50 would likely disadvantage women who have greater proportions of BC diagnosed younger than age 50 and may partially explain the higher proportions of advanced BC at diagnosis noted among many younger women of race and ethnicities other than White in this study. The decision by the US Preventive Services Task Force to lower the age of breast screening initiation from 50 to 40 was in part to address inequities around earlier age at diagnosis in an attempt to mitigate disparities in BC mortality by race/ethnicity.^29,30^

Although the overall ASMR for Black women in Canada was not significantly different than that for White women, Black women aged 40-49 had a 1.4 times higher age-specific BC mortality relative to White. Black Canadian women had a slightly lower median age of death (61) than Black women in the United States (63), while the median age of death in White Canadian women was slightly higher (71) than in the United States (70).^26^ These mortality differences could be related to the observed earlier peak age of diagnosis, which may mean BC cases are being diagnosed before screening is available, perhaps accounting for our observation that almost half of the stage III or IV BC cases among Black women were diagnosed before the age of 50 compared to one-fifth of stage III or IV cases among White women. However, given that Chinese and South Asian groups had a similar earlier peak age at diagnosis with no comparable increase in BC mortality, there are likely other factors involved.^5,31,32^ In Black women, over half of all BC cases diagnosed are molecular subtypes with poorer outcomes, as compared to about one-third of cases among White women. TN BC composed 20.5% of cases among Black women, and 9.5% among White women, mirroring the 19% and 9% seen in the United States.^26^ The 5-year survival for stage III TN BC is only 47%, compared with 89% for stage III LA BC, while stage I TN BC has a 5-year survival of 96%, on par with the 100% for LA.^25^ The greater than doubled proportion of TN BC among Black women, especially when diagnosed at a later stage, likely contributed to increased mortality. Furthermore, even in the setting of good prognosis LA, outcomes among Black women have been noted to be worse than among White women, which may be due to decreased efficacy of endocrine therapy among Black women.^33^

BC mortality rates among First Nations and Métis women aged 60-69 were higher compared to White women. Decreased screening participation may be contributory as observed in a study in Manitoba, where only 37% of First Nations women were up to date on mammographic screening compared to 59% of non-Indigenous women.^34,35^ However, if increased BC mortality was strictly due to inadequate screening, mortality would be expected to be increased across all ages greater than 50. Poorer BC outcomes among First Nations and Métis women are likely due to a complex interplay of biological, lifestyle, cultural, social, and system factors, which may create disparities in accessing care throughout the cancer continuum, from screening, to timely diagnosis and treatment.^36,37^

Our study had several limitations. As information on race/ethnicity is not collected in the CCR, this study included only cases that were linked to the census or NHS. Stage and subtype data could not be determined for all cases. Documentation of race/ethnicity for all cancer cases in Canada would improve future research on this topic. Most other race/ethnicity groups have younger population structures than the White population, which may result in more BC diagnoses at younger ages. We were unable to determine access to screening and treatment to establish their contribution to BC mortality. The strengths of this study are its large size: 47 000 BC cases (14 700 with stage information and 12 000 with BC molecular subtypes for 6 race/ethnicity groups) and 18 200 BC deaths. As well, the use of age-specific BC mortality rates allowed disaggregated estimates of various race/ethnicity groups including for ages 40-49 not currently covered by routine mammography screening.

Compared to White women, women from other race/ethnicity groups generally had an earlier peak age diagnosis of BC with younger age at diagnosis, were more likely to have an advanced stage of cancer at diagnosis, and often had molecular subtypes with poorer outcomes. At the same time, many race/ethnicity groups displayed a lower BC mortality than White women despite younger age at diagnosis. These findings suggest that caution should be used in extrapolating older evidence for screening efficacy, which was based on primarily White populations. Higher BC mortality in Black, Métis, and First Nations women suggests an examination of potential barriers to health care across the cancer care continuum may be merited for these women in order to decrease apparent inequities. Screening and health care policies may be well served to consider race/ethnicity-based differences in BC outcomes to advance health equity in Canada.

## Data Availability

Data is available publicly through the Reseach Data Centres program at Statistics Canada https://www.statcan.gc.ca/en/microdata/data-centres.
