# Correction to: Breast cancer incidence and mortality, by age, stage and molecular subtypes, by race/ethnicity in Canada

**DOI:** 10.1093/oncolo/oyag056

**Published:** 2026-03-02

This is a correction to: Anna N Wilkinson, Carmina Ng, Larry F Ellison, Jean M Seely, Breast cancer incidence and mortality, by age, stage and molecular subtypes, by race/ethnicity in Canada, *The Oncologist*, 2024, oyae283, https://doi.org/10.1093/oncolo/oyae283

The following changes have been made to the originally published paper. In the section **Interpretation**, text at the beginning of the second sentence is emended to read: “Among the 6 groups with sufficient cases staged, Black, Filipina, First Nations, and South Asian women[…] instead of: "Among the 6 groups with sufficient cases staged, Black, Filipina, First Nations, Métis, and South Asian women[…]”.

